# Heterogeneous Expression of T-type Ca^2+^ Channels Defines Different Neuronal Populations in the Inferior Olive of the Mouse

**DOI:** 10.3389/fncel.2016.00192

**Published:** 2016-08-04

**Authors:** Paolo Bazzigaluppi, Marcel T. G. de Jeu

**Affiliations:** Department of Neuroscience, Erasmus Medical CenterRotterdam, Netherlands

**Keywords:** inferior olive, calcium channels, subthreshold oscillations, membrane potential, synchrony

## Abstract

The neurons in the inferior olive express subthreshold oscillations in their membrane potential. This oscillatory activity is known to drive synchronous activity in the cerebellar cortex and plays a role in motor learning and motor timing. In the past years, it was commonly thought that olivary neurons belonged to a unique population of oscillating units and that oscillation properties were exclusively dependent on network settings and/or synaptic inputs. The origin of olivary oscillations is now known to be a local phenomenon and is generated by a combination of conductances. In the present work, we show the existence of at least two neuronal populations that can be distinguished on the basis of the presence or absence of low-voltage activated Ca^2+^ channels. The expression of this channel determines the oscillatory behavior of olivary neurons. Furthermore, the number of cells that express this channel is different between sub nuclei of the inferior olive. These findings clearly indicate the functional variability within and between olivary sub nuclei.

## Introduction

The inferior olive (IO) receives both motor and sensory information from the cerebellar nuclei and the body and provides one of the major afferents to the cerebellum: the climbing fiber (Desclin, 1974; Courville and Faraco-Cantin, 1978). It is well known that IO neurons express oscillations in their membrane potential both *in vivo* and *in vitro* (Llinas and Yarom, 1986; Chorev et al., 2007; Khosrovani et al., 2007) and it has been hypothesized that these oscillations control the timing of motor performances and motor learning (Welsh et al., 1995; Van Der Giessen et al., 2008). Lesions in the IO are known to cause severe motor abnormalities (Llinas et al., 1975; Horn et al., 2013), but the precise role IO rhythms play for motor behavior still needs to be elucidated. Low-Voltage Activated Ca^2+^ channels (LVA) have been implicated in the generation of physiological and pathophysiological rhythms in neurons (Huguenard, 1996; McCormick and Bal, 1997; Kim et al., 2001). During an hyperpolarizing event LVA Ca^2+^ channels will be de-inactivated and after termination of the hyperpolarization (i.e., anodal break), all available LVA Ca^2+^ channels can be activated to induce a Low-Threshold Ca^2+^ Spike (LTS; Crunelli et al., 1989; Perez-Reyes, 2003). These channels are involved in the generation of neuronal oscillations, resonance, and pacemaker activities (Huguenard, 1996; McCormick and Bal, 1997; Kim et al., 2001) and they are highly expressed in the olivo-cerebellar system (Talley et al., 1999), including neurons of the IO, Purkinje cells, and neurons of the deep cerebellar nuclei (Wolpert et al., 1998; Talley et al., 1999). Physiological and pharmacological studies suggested that LVA Ca^2+^ channels play a role in the control of intrinsic oscillatory properties of IO neurons (Llinas and Yarom, 1986; Lampl and Yarom, 1997). There are three isoforms of LVA Ca^2+^ channels and the IO expresses mainly the Ca_V_3.1 isoform (McKay et al., 2006). The presence of Ca_V_3.1 channels determines the physiological function of olivary neurons and the occurrence of subthreshold oscillations (Park et al., 2010; Zhan and Graf, 2012).

Although in many reports the implicit suggestion is made that the IO consist of a uniform population of oscillating units, there are results showing that subthreshold oscillations are not a phenomenon common to all olivary neurons (Manor et al., 1997; Chorev et al., 2006; Khosrovani et al., 2007). In order to understand the function of the IO it is necessary to know which olivary neurons oscillate and why. In this study, we hypothesize that the expression level of the LVA Ca^2+^ channels Ca_V_3.1 determines the oscillatory behavior of olivary neurons and that this channel is heterogeneously expressed throughout the IO.

To examine whether the oscillatory behavior of olivary neurons depends on the expression level of Ca_V_3.1, we determined the electrophysiological behavior of olivary neurons in relation to the expression of Ca_V_3.1 channel by combining whole-cell electrophysiology with immunohistochemistry. Our results reveal that olivary neurons can be distinguished in two populations based on the presence or absence of Ca_V_3.1 channels in their membranes and their capability of generating spontaneous (or induced) subthreshold oscillations in their membrane potentials. These novel observations provide the experimental ground both for previous model studies (Manor et al., 1997) and for the design of future virtual networks of the Olivo Cerebellar system.

## Materials and Methods

### *In vitro* Electrophysiology and Slices Preparation

Brains of C57Bl6 mice (age: 3- to 4-weeks) were removed from their skull after decapitation and were placed in ice-cold artificial cerebrospinal fluid (ACSF). The brains were trimmed to a block containing the brainstem and coronal slices of 200 μm were cut with a vibroslicer (Leica VT1000). The slices were transferred to a storage chamber filled with ACSF containing (in mM): 124 NaCl, 5 KCl, 1.25 Na_2_HPO_4_, 2.5 MgSO_4_, 2 CaCl_2_, 26 NaHCO_3_, and 20 _D_-glucose, bubbled with 95% O_2_ and 5% CO_2_ (all chemicals were purchased from Sigma–Aldrich). The experiments were all in accordance with the Dutch national guidelines on animal experiments. And as required by Dutch legislation, the experiments were approved by the institutional animal welfare committee (DEC, Erasmus MC, Rotterdam, The Netherlands).

Whole-cell patch-clamp recordings were performed at room temperature (Voltage Clamp experiments) or at more physiological temperatures (34–35°C, Current Clamp experiments). Patch pipettes were pulled with a glass electrode puller (P-92, Sutter instruments) using borosilicate glass (Sutter instruments). Electrode resistances varied between 4 and 6 MΩ.

For *Voltage Clamp* recordings, pipettes were filled with recording solution containing (in mM): 110 CsCl, 1 CaCl_2_, 5 MgCl_2_, 10 EGTA, 10 HEPES, 4 Na_2_ATP, 15 phosphocreatine, 0.2 Alexa hydrazide 488, and 1 QX-314 (pH 7.3; osmolarity, 305 mOsm). To isolate Ca^2+^ currents, the following drugs were added to the extracellular solution (in mM): 10 tetraethylammonium chloride (TEA), 1 4-aminopyridine (4-AP), 1 tetrodotoxin (TTX), and 2 CsCl. For *Current Clamp* recordings pipettes were filled with recording solution containing (in mM): 120 K gluconate, 9 KCl, 10 KOH, 3.48 MgCl_2_, 4 NaCl, 10 HEPES, 4 Na_2_ATP, 0.4 Na_3_GTP, 0.2 Alexa hydrazide 488, and 17.5 sucrose; pH was adjusted to 7.25.

Membrane voltages and currents were recorded using an EPC-10 amplifier (filtered at 10 kHz and digitized at 25 kHz; HEKA electronics, Germany) and relayed to a personal computer equipped with Pulse software (HEKA electronics, Germany). During voltage clamp recordings, at least 70% of the cell capacitance and series resistance were compensated and automatic leak subtraction was applied on-line (using hyperpolarizing pulses with time constant of 100 μs and amplitude one third of the injected depolarizing step). Voltage and Current clamp measurements were corrected for a junction potential of -5 and -8 mV, respectively. Input resistance (*R*_i_) was measured by injection of hyperpolarizing test currents (200 pA; 100 ms) and was calculated from the voltage transient toward the end of current injection. Recordings were excluded if the input resistance varied by more than 15%.

3,5 -dichloro - N- [1- (2,2 - dimethyl -tetrahydro-pyran-4-ylmethyl)-4-fluoro-piperidin-4-ylmethyl]-benzamide (TTA-P2) was bath applied to block the Ca_V_3.1 channels (Dreyfus et al., 2010). TTA-P2 was kindly provided by Victor N. Uebele (Merck Research Laboratories, West Point, PA, USA). TTA-P2 was prepared as a 10 mM stock solution in dimethylsulfoxide (DMSO) and kept at -20°C until use. TTA-P2 was used in a final concentration of 1 μM, therefore that the final concentration of DMSO was ∼0.01% (too low to have a significant effect on neuronal physiology). Data was analyzed in Clampfit 9.2 (Axon Instruments, Foster City, CA, USA).

### Immunohistochemistry and Confocal Imaging

After the electrophysiological recording, slices were fixed for 1 h in 4% paraformaldehyde (PFA) solution and then stored at 4°C in Phosphate Buffer (PB, 0.1 M). Slices were washed twice with PB (0.1 M) and the tissue was incubated in normal horse serum (10%) and triton-X100 (0.5%) for 2 h. To visualize Ca_V_3.1 channels and the neuronal nuclei, slices were incubated for 96 h with rabbit anti-Ca_V_3.1 antibody [Chemicon, final dilution 1:1500, (Lee et al., 2008)] and with goat NeuN antibody (Chemicon, final dilution 1:20000). After incubation with the primary antibody, IO slices were first rinsed four times for 10 min in PB (0.1 M) then incubated for 3 h with anti-goat Cy3 and anti-rabbit Cy5 conjugated antibodies (Jackson Immunoresearch, USA; final dilution 1:200). To validate the specificity of anti-Ca_V_3.1 antibody, we applied the above mentioned procedure on brain slices of Cav3.1 -/- mutant mice and wild-type mice [i.e., control; Supplementary Figure S1, see also (Lee et al., 2008)]. The recorded neuron was identified by the fluorescence signal of Alexa 488 hydrazide. To investigate the Ca_V_3.1 expression in the recorded neuron, confocal images of slices were made using a Zeiss LSM700 confocal laser scanning microscope operated by Zen 2009 software (Carl Zeiss, Germany).

To measure Ca_V_3.1 expression throughout olivary sub nuclei, we counted Ca_V_3.1 positive and negative stained neurons in the thin slices. For these experiments, two mice were transcardially perfused with 4% PFA, the brain was extracted, embedded in gelatin and cryo-sliced in slices of 40 μm (from now on these slices will be referred to as *thin slices*). These thin slices were stained immunohistochemically for Ca_V_3.1 in the same way as described above. Olivary neurons were counted based on the following criteria: superficial location and an intact nucleus (selected on the base of neat and round NeuN signal). A total number of 52 thin slices were analyzed; the medial accessory olive (MAO) was found in 50 slices, the dorsal accessory olive (DAO) in 31 slices, the principal olive (PO) in 27 slices, the beta nucleus (Beta) in 18 slices and the dorsal cap of Kooij (IOK) in 10 slices. Olivary neurons expressing a Ca_V_3.1 signal two times higher than the background were counted as immuno-positive. Due to technical difficulties, we were not able to quantitatively assess the expression level of Ca_V_3.1 channels in a continuous gradual scale. For this reason olivary neurons were binomially considered immuno-positive or immuno-negative. Statistical comparisons between the percentages of immuno-positive neurons in five subnuclei of the IO were performed in SPSS environment using a one-way ANOVA and three different *post hoc* tests (Tukey HSD, Bonferroni and LSD). Given the consistency of the results between the three used tests, we present only the *p*-values of the Tukey HSD test.

## Results

### Ca_V_3.1 Channels Are Unevenly Distributed between Olivary Neurons

**FIGURE 1:**
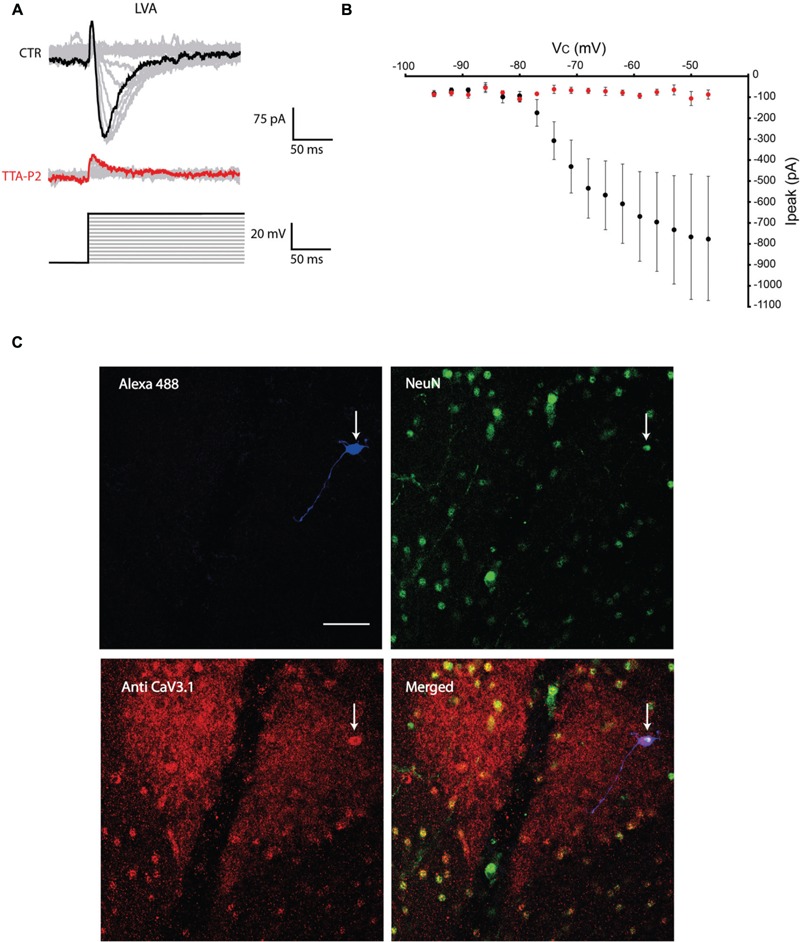
**Olivary neurons that express low-voltage activated (LVA) Ca^2+^ currents are immuno-positive for Ca_V_3.1 channels. (A)** LVA Ca^2+^ currents are evoked by depolarizing the olivary neuron upto -47 mV from an holding potential of -100 mV under control conditions (black and gray traces, CTR) and after 5 min of diffusion with 1 μM TTA-P2 (red and gray traces, TTA-P2). **(B)** I-V relationship of LVA Ca^2+^ currents recorded from olivary neurons under control conditions (black dots) and after 5 min of diffusion of 1 μM TTA-P2 (red dots). TTA-P2 blocks the LVA Ca^2+^ current (*p* = 0.02, *n* = 9, paired Student *t*-test). **(C)** Panels, images of the olivary neuron recorded in **(A).** Top left panel, image of the recorded neuron obtained from the Alexa 488 signal (blue). Top right panel, image obtained from a NeuN staining (green). Bottom left panel, image of Ca_V_3.1 channels in the IO (red). Bottom right panel, merged image of the other three panels. White arrow indicates the recorded neuron. Scale bar: 50 μm.

**FIGURE 2:**
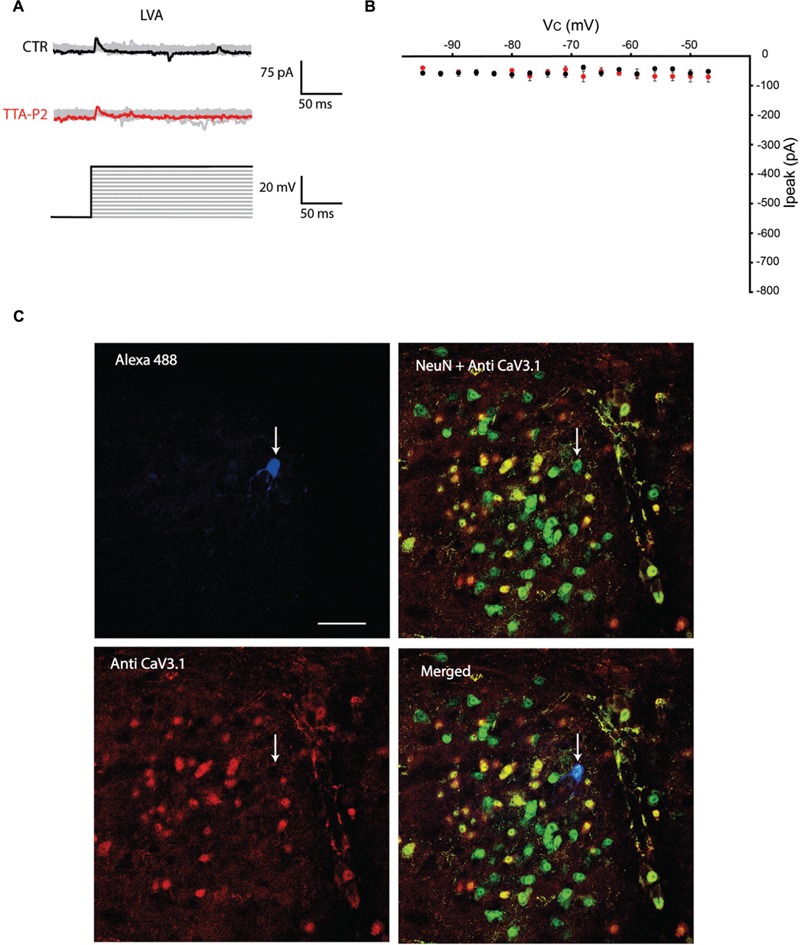
**Olivary neurons that do not express LVA Ca^2+^ currents are immuno-negative for Ca_V_3.1 channels. (A)** Same protocol as in **Figure 1A**, the depolarizing step does not evoke LVA Ca^2+^ currents in this olivary neuron (black and gray traces, CTR) and is insensitive to 1 μM TTA-P2 (red and gray traces, TTA-P2). **(B)** I–V relationship of olivary neurons without a LVA Ca^2+^ current under control conditions (black dots) and after 5 min of diffusion with 1 μM TTA-P2 (red dots). These conditions are not significantly different (*p* = 0.63, *n* = 7, paired Student *t*-test). **(C)** Panels, images of the olivary neuron recorded in **(A)**. Top left panel, image of the recorded neuron obtained from the Alexa 488 signal (blue). Top right panel, image of Ca_V_3.1 channels in the IO (red). Bottom left panel, merged image of Alexa 488, NeuN (green) and Ca_V_3.1 antibody signals. Bottom right panel, merged image of NeuN and Ca_V_3.1 antibody signal. White arrow indicates the recorded neuron. Scale bar: 50 μm.

The first series of electrophysiological experiments were performed in Voltage Clamp mode and allowed us to identify two distinct populations of olivary neurons based on the presence of LVA Ca^2+^ currents. An example of a neuron that expresses LVA Ca^2+^ currents is shown in **Figure 1** and a neuron that does not express LVA Ca^2+^ currents is shown in **Figure 2**. These experiments aimed to isolate the LVA Ca^2+^ currents (see Materials and Methods section for experimental conditions) leaving the high voltage activated (HVA) Ca^2+^ current unaltered. LVA Ca^2+^ currents were evoked by 200 ms voltage steps to depolarizing potentials ranging from -95 to -47 mV following an holding potential of -100 mV (i.e., hyperpolarizing pre-pulse of 1 s, **Figures 1A** or **2A**). Neurons expressing LVA Ca^2+^ currents (9 out of 16: 56% **Figure 1A**) showed a transient current that reached a peak value of 774.6 ± 90.4 pA (*n* = 9), when a voltage step was made to -47 mV. This current was potently blocked by 1 μM TTA-P2 (87.2 ± 11.7 pA, **Figure 1B**, *p* = 0.02, *n* = 9, paired Student *t*-test). Neurons expressing no LVA Ca^2+^ currents (7 out of 16: 44%, **Figure 2A**) showed an average current of 68.7 ± 7.1 pA (*n* = 7) when a voltage step was made to -47 mV. This value is close to the holding current of olivary neurons at -60 mV (60–70 pA). Moreover, diffusion of 1 μM TTA-P2 in the bath did not significantly reduce this value which settled to 58.5 ± 4.6 pA (**Figure 2B**, *p* = 0.63, *n* = 7, paired Student *t*-test).

Further depolarizations of the membrane potential up to 0 mV induced the activation of the HVA Ca^2+^ currents in both populations of neurons. In LVA positive neurons, the action of TTA-P2 on LVA did not significantly reduce the amplitude of the HVA current (peak amplitude at -10 mV, before TTA-P2: 3145.3 ± 118.1 pA vs. after TTA-P2: 2409.1 ± 109.6 pA, *p* = 0.07, *n* = 5, paired Student *t*-test). Also in LVA-negative neurons the amplitude of the HVA current remained unchanged after TTA-P2 diffusion (before TTA-P2: 1488.5 ± 102.4 pA vs. after TTA-P2: 960.2 ± 120.1 pA, *p* = 0.34, *n* = 5, paired Student *t*-test). However, in both cases we observed a trend that 1 μM TTA-P2 partially block the HVA current. Furthermore, it is noteworthy that LVA-positive neurons expressed significantly larger peak amplitude of HVA currents (3145.3 ± 84.1 pA) than LVA-negative ones (1488.5 ± 102.4 pA) under control conditions (*p* = 0.004, *n* = 5, unpaired Student *t*-test, see Discussion).

All the olivary cells were stained during the recordings with Alexa hydrazide 488 and the electrode was slowly retracted at the end of the recording to leave the neuron intact and in place. The slices were then fixated and immunohistochemistry was performed to stain for neuronal nuclei [NeuN, (Mullen et al., 1992)] and Ca_V_3.1 channels (see Materials and Methods section for detailed procedures). All the recorded cells were NeuN-positive, confirming that they were indeed neurons. Moreover, the LVA Ca^2+^ channel expression revealed by the Ca_V_3.1 antibody matched with the electrophysiological profile: olivary neurons that expressed LVA Ca^2+^ currents were also immuno-positive to the Ca_V_3.1 antibody (*n* = 9, **Figure 1C**), whereas LVA-negative neurons were immuno-negative for the Ca_V_3.1 antibody (*n* = 7, **Figure 2C**; *p* < 0.01, *n* = 16, χ^2^ test).

### The Presence of Ca_V_3.1 Channels Determines Oscillatory Profile of Olivary Neurons

**FIGURE 3:**
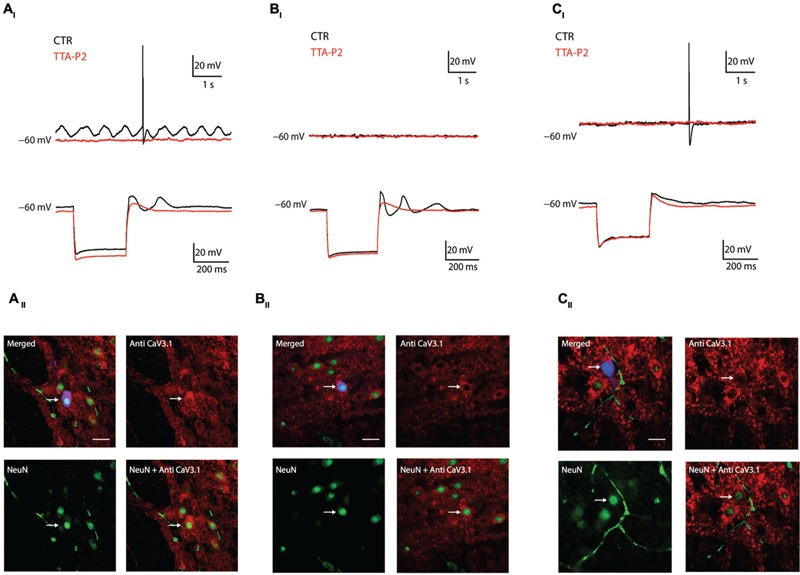
**Combined current clamp recordings and immunohistochemistry of olivary neurons. (A_I_)** Top traces: spontaneous oscillations (black trace) are blocked by 1 μM TTA-P2 (red trace). Bottom traces: rebound-depolarization and -oscillation are blocked by 1 μM TTA-P2 (black vs. red trace). **(A_II_)** Panels: spontaneous oscillators are immuno-positive for Ca_V_3.1 channels. Top left panel, merged image of Alexa 488 (blue), NeuN (green) and Ca_V_3.1 antibody (red) signals. Top right panel, image of Ca_V_3.1 channels in the IO. Bottom left panel, image obtained from a NeuN staining. Bottom right panel, merged image of NeuN and Ca_V_3.1 antibody signals. **(B_I_)** Example of conditional oscillator. Top traces: membrane potential without subthreshold oscillation under control conditions (black trace) and after diffusion of 1 μM TTA-P2 (red trace). Bottom traces: rebound-depolarization and -oscillation are blocked by 1 μM TTA-P2 (black vs. red trace). **(B_II_)** Panels: conditional oscillators are immuno-positive for Ca_V_3.1 channels. Top left panel, merged image of Alexa 488 (blue), NeuN (green), and Ca_V_3.1 antibody (red) signals. Top right panel, image of Ca_V_3.1 channels in the IO. Bottom left panel, image obtained from a NeuN staining. Bottom right panel, merged image of NeuN and Ca_V_3.1 antibody signal. **(C_I_)** Example of a non-oscillating neuron. Top traces: membrane potential without subthreshold oscillation under control conditions (black trace) and after diffusion with 1 μM TTA-P2 (red trace). Bottom traces: neuron does not express rebound oscillations (black trace). Rebound-depolarizations are not blocked by 1 μM TTA-P2 (black vs. red trace). **(C_II_)** Panel: non-oscillating neurons are immuno-negative for Ca_V_3.1 channels. Top left panel, merged image of Alexa 488 (blue), NeuN (green), and Ca_V_3.1 antibody (red) signals. Top right panel, image of Ca_V_3.1 channels in the IO. Bottom left panel, image obtained from a NeuN staining. Bottom right panel, merged image of NeuN and Ca_V_3.1 antibody signal. White arrows indicate the recorded neurons. Scale bar: 60 μm.

The previous experiment showed that the presence of LVA is linked to Ca_V_3.1 channels expression, we then hypothesize that oscillations in the IO depend on Ca_V_3.1 expression. In the following series of experiments, we recorded olivary neurons in Current Clamp mode (*n* = 18) and correlated their oscillatory activity with their immuno-reactivity to Ca_V_3.1 antibody. We could identify three different oscillation profiles which match the model of Manor et al. (1997) and Chorev et al. (2006) (see Discussion). We were able to record neurons that spontaneously presented sinusoidal subthreshold oscillations in their membrane potential (4 out of 18: 22%), as already showed by others *in vitro* (Llinas and Yarom, 1986; Devor and Yarom, 2002b) and *in vivo* (Khosrovani et al., 2007; Bazzigaluppi et al., 2012a). The spontaneous sinusoidal oscillations of these neurons were blocked by 1 μM TTA-P2 and immunohistochemistry revealed the presence of Ca_V_3.1 channels in these neurons (**Figures 3A_I,II_**). Besides the spontaneously oscillating units, we also recorded neurons that lacked oscillatory behavior during resting conditions, but responded with a rebound low threshold spike (LTS) followed by a transient sinusoidal oscillation after the termination of an hyperpolarizing current (7 out of 18: 39%). The LTS and the induced sinusoidal oscillations of these neurons were also blocked by 1 μM TTA-P2, indicating the importance of LVA Ca^2+^ currents for these subthreshold responses (**Figure 3B_I_**). These neurons also expressed Ca_V_3.1 channels (**Figure 3B_II_**). Ultimately, we recorded neurons that lacked oscillatory behavior during rest conditions, and responded after the anodal break with a small rebound LTS that was not followed by the transient sinusoidal oscillations (7 out of 18: 39%). These neurons were insensitive to 1 μM TTA-P2 and were all immuno-negative for Ca_V_3.1 channel antibody (**Figures 3C_I,II_**).

**FIGURE 4:**
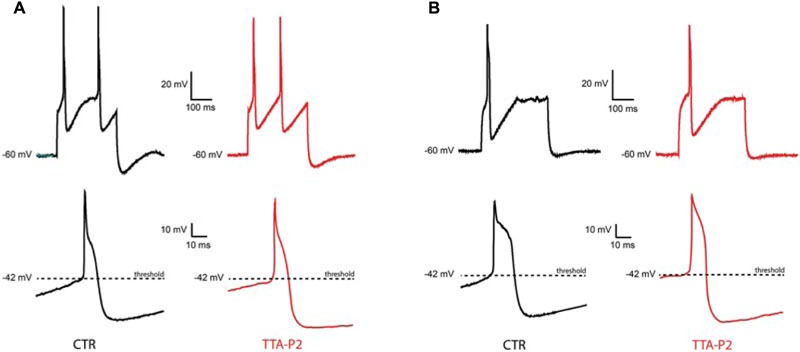
**Sensitivity of suprathreshold activity to Ca_V_3.1 blocker TTA-P2. (A)** A conditional oscillator responds to a 200 ms depolarizing step with action potential in control (top left, CTR) as well as after TTA-P2 (top right, TTA-P2), the threshold of the AP remains unchanged before (bottom left, CTR) and after (bottom right, TTA-P2) TTA-P2. **(B)** Silent neurons respond to a 200 ms depolarizing step with action potential in control (top left, CTR) as well as after TTA-P2 (top right, TTA-P2), the threshold of the AP remains unchanged before (bottom left, CTR) and after (bottom right, TTA-P2) TTA-P2; for quantitative description of the analyzed parameters see **Table 1**.

To check whether the TTA-P2 is affecting the excitability and the supra-threshold activity of olivary neurons, we compared the APs before and after the application of 1 μM TTA-P2 (**Figure 4**). Quantitative comparison of action potential characteristics (AP peak amplitude, threshold, number of APs per train, half peak time, half width time, number of spikelets, AHP peak amplitude; see **Table 1**) were performed on the first action potential evoked by a 400 pA depolarizing step from a holding potential of -60 mV. Because spontaneous oscillations have a disturbing effect on the above mentioned analysis, we analyzed only neurons that generated sinusoidal oscillations after an anodal break and neurons that did not express sinusoidal oscillations. In both, the conditioned oscillating and the non-oscillating neurons, not one of these parameters was significantly different before or after the incubation of the slices in 1 μM TTA-P2 (see **Table 1** for details). These results are comparable with the results obtained in the thalamus by Dreyfus et al. (2010). Therefore, these neurons have no different spike waveforms and TTA-P2 does not affect the conductances responsible for guiding the suprathreshold activity of olivary neurons.

**Table 1:** Comparison of the AP parameters of conditional oscillators (c.o., left part) before and after TTA-P2, non-oscillating neurons (n.o., middle part) before and after TTA-P2 and between c.o. and n.o. before TTA-P2 (right part, c.o. vs. n.o.).

	Conditional oscillators (c.o.)			Non-oscillating (n.o.)			c.o. vs. n.o.
	Before TTA-P2	After TTA-P2	*p*-value (*n* = 6)	Before TTA-P2	After TTA-P2	*p*-value (*n* = 7)	*p*-value
AP peak amplitude (mV)	61.8 ± 1.2	59.6 ± 1.5	0.19	57.7 ± 1.2	57.0 ± 0.7	0.441	0.93
Number of APs	2.0 ± 0.1	1.5 ± 0.1	0.08	2.2 ± 1.6	2.0 ± 0.1	0.21	1.00
Half-peak time (ms)	0.4 ± 0.2	0.5 ± 0.1	0.63	0.7 ± 0.1	0.6 ± 0.1	0.30	0.15
Half-width time (ms)	5.3 ± 1.7	4.8 ± 0.5	0.55	4.7 ± 0.6	4.3 ± 0.4	0.39	0.23
# spikelets	2.6 ± 0.2	2.3 ± 0.2	0.17	2.5 ± 0.1	2.5 ± 0.1	0.50	0.66
AHP peak (mV)	33.9 ± 1.8	25.5 ± 1.2	0.11	29.0 ± 0.5	27.3 ± 0.1	0.38	0.28

### Expression of Ca_V_3.1 Channels in Olivary Subnuclei

**FIGURE 5:**
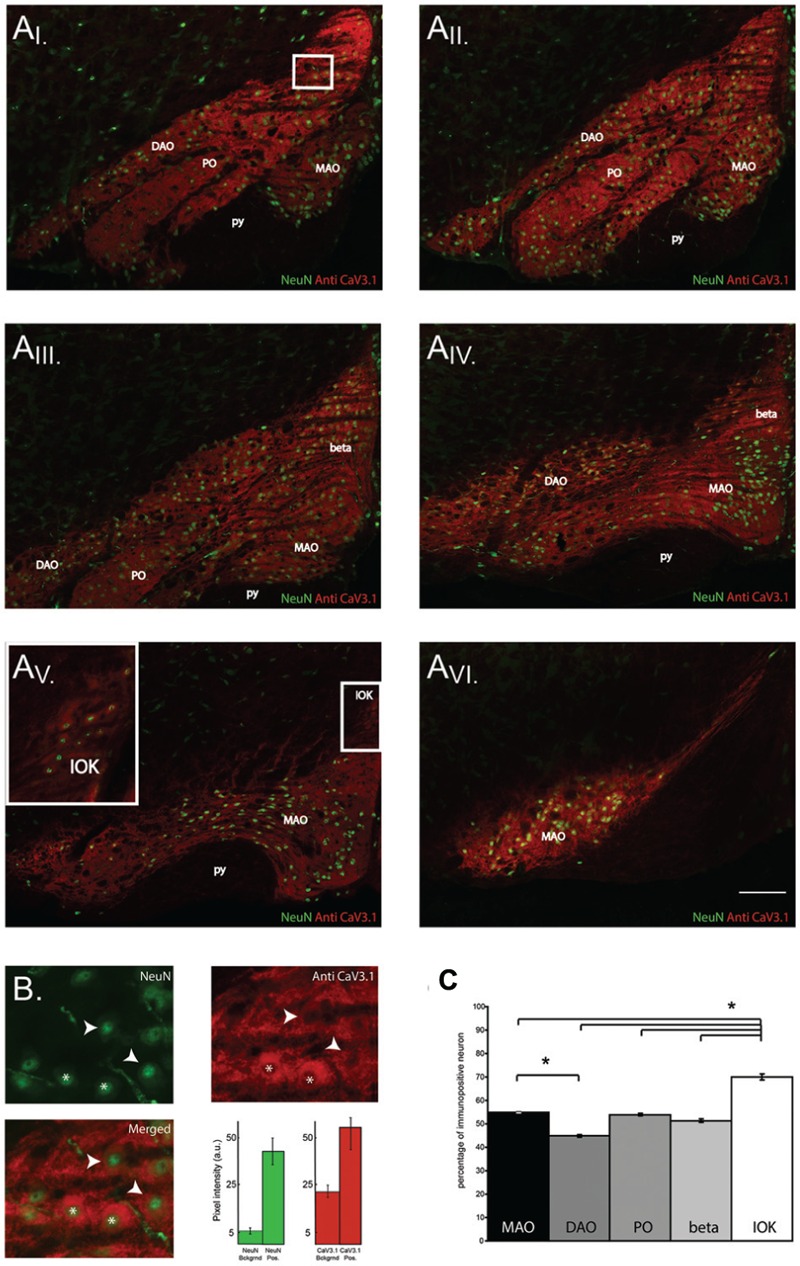
**Ca_V_3.1 channel expression in olivary subnuclei. (A)** Immunohistochemistry on transversal hemi-slices of the Inferior Olive (inferior olive). **(A_I-V I_)** Rostro-caudal arrangement (top-left to bottom right) of olivary slices with an inter-slice distance of 200 μm. Olivary subnuclei are indicated in the images and are the Medial accessory olive (MAO), the Principal Olive (PO), the Dorsal accessory olive (DAO), the beta nucleus (beta) and the Dorsal cap of Kooij (IOK). py: pyramidal tract. **(B)** Magnified area of the white square in the **(A_I_)** panels: example of immunohistochemistry of olivary neurons. Top-left panel, image obtained from a NeuN staining (green). Top right panel: image of Ca_V_3.1 channels (red) in the IO. Bottom left panel, merged image of NeuN and Ca_V_3.1 antibody signals. Arrow-heads indicate neurons considered immuno-negative for Ca_V_3.1 channels, whereas asterisks indicate neurons immuno-positive for Ca_V_3.1 channels. Histograms show the pixel intensity of the background signals and of the NeuN (green) and Ca_V_3.1 signal (red) of positively stained neurons from the slice presented in **(B)**. Error bars: mean ± SD. **(C)** Comparison between the percentages of neurons immuno-positive for Ca_V_3.1 channels of the different olivary subnuclei, MAO presents more immuno-positive neurons than DAO, IOK contains more immuno-positive neurons than all the other subnuclei (one-way ANOVA: MAO, *p* = 0.02; DAO, *p* = 0.01; PO, *p* = 0.02; beta, *p* = 0.009). The analysis was performed on slices cut every 40 μm. Scale bar: 100 μm.

The results shown so far demonstrate that Ca_V_3.1 negative olivary neurons are not capable of generating LVA currents and do not express subthreshold oscillations. However, the number of recorded neurons in VC and CC experiments is relatively small compared to the whole neuronal population of the IO. For this reason, we were not able to determine whether olivary subnuclei were expressing different percentages of Ca_V_3.1 channel positive neurons. Immunohistochemistry experiments were performed on thin slices to obtain information about the distribution of Ca_V_3.1 channel positive and negative neurons in the different subnuclei of the IO. In this experiment, we identified and analyzed five subnuclei of the IO. We counted the amount of Ca_V_3.1 channel immuno-positive and negative neurons in the DAO, the MAO, the PO, the beta nucleus and in the IOK as shown in **Figure 5A**. In these subnuclei, the percentage of immuno-positive neurons over the total of NeuN positive cells was calculated (**Figure 5B**, see Materials and Methods section). The expression of Ca_V_3.1 channel positive neurons is almost homogenous between different subnuclei (see below). In the DAO 44.8 ± 0.5% of neurons express Ca_V_3.1 channels and this value is significantly smaller than in the MAO (54.9 ± 0.2%, *p* = 0.02), but it is not smaller than in the PO (53.9 ± 0.5%) and beta nucleus (51.3 ± 0.5%). The IOK expresses the highest amount of Ca_V_3.1 channel positive neurons (70.2 ± 1.2%) and it is significantly higher than in all the other subnuclei (**Figure 5C**, MAO, *p* = 0.02; DAO, *p* = 0.01; PO, *p* = 0.02; beta, *p* = 0.009). These results show that in each olivary subnuclei there is a relevant percentage of neurons that do not express the Ca_V_3.1 channel at a detectable level and therefore are most likely not able to generate subthreshold oscillations, neither spontaneous or induced after an hyperpolarization (conditional oscillator). Despite the significant difference between DAO and the MAO, the amount of Ca_V_3.1 positive neurons in the MAO, PO, DAO, and beta is around 50%. Therefore, these subnuclei will grossly express the same proportions of oscillating neurons. The IOK has a significant higher number of neurons expressing Ca_V_3.1 channels than the other subnuclei and should therefore contain a much higher proportion of oscillating neurons.

Overall, the present work demonstrates that the expression level of Ca_V_3.1 channels in olivary neurons varies substantially throughout the IO. Immuno-positive neurons to the antibody against Ca_V_3.1 channels express a T-type Ca^2+^ current, which is sensitive to the blocker of the Ca_V_3.1 family, TTA-P2. Immuno-negative neurons failed to show the T-type Ca^2+^ current. In our current clamp experiments, two oscillatory and one non-oscillatory state were identified within the population of olivary neurons. Immuno-positive neurons show either spontaneous sinusoidal oscillations like the ones observed *in vivo* (Chorev et al., 2007; Khosrovani et al., 2007; Bazzigaluppi et al., 2012b) and *in vitro* (Llinas and Yarom, 1981, 1986; Devor and Yarom, 2002a; Long et al., 2002) or the anodal-break induced sinusoidal oscillations (i.e., conditional oscillator). Both these oscillations were blocked by TTA-P2. Immuno-negative neurons do not express any of the described oscillatory profiles and were insensitive to TTA-P2. Further immunohistochemical analyses showed that immuno-positive and immuno-negative neurons were not equally distributed throughout the different subnuclei of the IO.

## Discussion

The present work shows that olivary neurons can be divided into at least three different populations based on their subthreshold oscillatory profile or two based on their Ca_V_3.1 channels expression. In Ca_V_3.1 immuno-positive neurons, 1 μM TTA-P2 blocked spontaneous subthreshold oscillations and conditional oscillations as well as their underlying LVA current. Although, Choe et al. (2011) showed that TTA-P2 can block HVA currents, the sensitivity for LVA currents is a 100-fold higher. Our voltage Clamp experiments might suggest that 1 μM TTA-P2 is partially able to block HVA currents (both results NS), but no consequences on the width of the spike afterdepolarization is observed in our Current Clamp experiments (**Table 1**). It is therefore most likely that blocking the subthreshold oscillations by 1 μM TTA-P2 is due to the complete block of the LVA current and not to a partial block of the HVA current. In addition to the confirmation by anti-Ca_V_3.1 immunohistochemistry, there are several other indications that the Ca_V_3.1 channel is the most likely candidate that is responsible for the LVA current in our IO neurons. First of all, IO neurons express both Ca_V_3.1 and Ca_V_3.3 channels, but the Ca_V_3.1 is more highly expressed throughout the entire IO than Ca_V_3.3 channels. The Ca_V_3.3 channels are only expressed in the caudal part of the IO with a much lower expression level (Talley et al., 1999). Secondly, although 1 μM TTA-P2 can block both Ca_V_3.1 and Ca_V_3.3 channels (Shipe et al., 2008), the electrophysiological features of our IO neurons that were treated with 1 μM TTA-P2 (i.e., lack of spontaneous oscillations, lack of conditioned oscillations, reduce size of rebound depolarization; **Figure 3**) are similar to the electrophysiological features observed in IO neurons of Ca_V_3.1 -/- mutant mice (Choi et al., 2010; Park et al., 2010).

From our results, it also emerges that HVA Ca^2+^ current is larger in immuno-positive than in negative Ca_V_3.1 neurons. Choi et al. (2010) already showed that HVA Ca^2+^ currents (i.e., via Ca_V_2.1 channels) and LVA Ca^2+^ currents (via Ca_V_3.1 channels) in IO neurons are important determining factors in the genesis and dynamic control of subthreshold oscillations in the IO. The lower levels of HVA Ca^2+^ currents in the Ca_V_3.1 negative neurons might correlate with the absence of the LVA Ca^2+^ current in order to maintain neuronal homeostasis. This result suggests that the different neuronal populations in the IO might optimize the ratio between these two conductances to fulfill the requirements of proper membrane physiology. Alternatively, the larger HVA response observed in the LVA positive neurons can be explained by a residual population of LVA channels which can still be activated at potential greater than -20 mV (Perez-Reyes, 2003).

In the last four decades olivary neurons have always been presented as an unique population of oscillating units (Llinas et al., 1975; Llinas and Yarom, 1981, 1986; Long et al., 2002; Placantonakis et al., 2006) overlooking or ignoring the possibility that non-oscillating neurons are also a functional set of olivary neurons. In Manor et al. (1997) and Chorev et al. (2006), the authors hypothesize the existence of four different types of “oscillators” in the IO and Chorev et al. (2006) confirmed their hypothesis using the dynamic clamp technique to change low-threshold conductances and drive the transitions between oscillatory states. Our data confirms the hypothesis of Manor et al. (1997) and forms the first biological indication of the existence of two different populations of neurons in the IO based on the expression of Ca_V_3.1 channels. However, the density of Ca_V_3.1 channels expression in the IO neurons will most likely have variable levels (i.e., high, moderate, and low), which can form the basis of the existence of two oscillatory states in our population of Ca_V_3.1 channel positive neurons; the spontaneous and conditional oscillators. We could not quantitatively measure the density of Ca_V_3.1 channels, but we hypothesize that high level of Ca_V_3.1 expression corresponds to spontaneous oscillators (i.e., **Figure 3A**), whereas neurons expressing intermediate densities (i.e., **Figure 3B**) constitute to conditional oscillators. The model of Manor et al. (1997) also revealed a fourth oscillatory state: the conditional bi-stable state. We never encountered a neuron that could be defined as being in the conditional bi-stable state. As also indicated by Chorev et al. (2006), the high input resistance necessary to reach the conditional bi-stable state is not likely to occur in a biological scenario, but can of course be implemented in the *in silico* model of Manor et al. (1997).

Our data show that the IO is equipped with spontaneous and conditional oscillators alongside with non-oscillating neurons. While it is plausible that spontaneous oscillators can drive conditional oscillating neighbors into a coherent oscillatory state (as observed *in vitro* by Leznik and Llinas, 2005) and consequently synchronizing their climbing fiber activity, it seems unlikely that they can do the same with the non-oscillating units. This is due to the small conductivity of the gap junctions (GJ) in the IO, which are constructed by Connexin36 proteins (Srinivas et al., 2006; Van Der Giessen et al., 2006). This small electrotonic coupling between olivary neurons might be able to synchronize but not to relay the oscillations. Moreover, synaptic inputs might also be able to drive the conditional oscillators into an oscillatory state. As the present data show, the termination of a prominent hyperpolarization [as the one exerted by the activation of the nucleo-olivary pathway (Bazzigaluppi et al., 2012b)] can temporarily induce sinusoidal oscillations in the conditional oscillators and therefore trigger coherent activity in clusters of coupled neurons.

It has been shown that the subthreshold oscillations determine the output of olivary neurons (Mathy et al., 2009; Bazzigaluppi et al., 2012a; De Gruijl et al., 2012). In this study, we could not find any difference in climbing fiber burst activity (measured by spikelet counts) between the two non-spontaneous oscillating units: the conditional oscillators and non-oscillating units. The absence of spontaneous oscillations might deprive the olivary neuron from the ability to modulate the climbing fiber burst (Mathy et al., 2009; Bazzigaluppi et al., 2012a). Therefore, the consequences of differential Ca_V_3.1 expression can also affect the output of the IO at the level of climbing fiber burst modulation (i.e., spikelets modulation).

The thin slice immunohistochemistry of Ca_V_3.1 indicates that approximately 50% of the neurons of the DAO, MAO, PO, and beta nucleus express Ca_V_3.1 at a level to generate subthreshold oscillation (either spontaneous or conditional oscillations). Remarkably, the other 50% of the neurons of the subnuclei will most likely not generate subthreshold oscillations. These results show the physiological variability, and consequently the functional variability, of olivary neurons within a subnucleus. The high number of Ca_V_3.1 expressing neurons in the IOK suggests a high proportion of oscillating neurons in this sub nucleus, which so far has not yet been found (Urbano et al., 2006). Further research on IOK neurons is necessary to clarify our putative differences with the findings of Urbano et al. (2006).

The intra-nuclear electrotonic network plays a fundamental role in the physiology of the IO. However, the subthreshold oscillations are an intrinsic property of olivary neurons and are generated by a specific combination of ion channels (Long et al., 2002; De Zeeuw et al., 2003), the coherence of these oscillations requires electrotonical coupling via GJs (Leznik and Llinas, 2005). To obtain more insight on how the IO works, it is important to know the composition of the olivary electrotonically coupled ensembles and the conditions that control the oscillatory state of single cells. Are the electrotonic coupled ensembles composed of a homogeneous or a heterogeneous population of neurons? And in the latter case, what is the ratio of these different oscillating and non-oscillating units within an IO ensemble? Do these different ensemble compositions generate different network properties? Although it is already known that input from the cerebellar nuclei can temporarily drive the transition of a spontaneous oscillating unit into a non-oscillating state by way of shunting (Bazzigaluppi et al., 2012b; Lefler et al., 2014), the mechanisms that control the density of Ca_V_3.1 expression might be important for the transition between different electrical behaviors on a longer timescale. New studies on the composition of these IO networks and the intriguing dynamics underlying transitional changes in electrical behavior will provide the necessary insights on the integrative and computational power of the IO.

In the present work, we investigated the electrophysiology and expression of the Ca_V_3.1 channel in olivary neurons. We used electrophysiological recordings from neurons in different olivary subnuclei combined with immunohistochemistry to show that two different neuronal populations can be distinguished on the base of their Ca_V_3.1 channel expression. Moreover, this factor determines the capability and the propensity of olivary neurons to spontaneously generate subthreshold oscillations. Two oscillatory and one non-oscillatory state were observed: spontaneous oscillators, conditional oscillators and non-oscillating units. Ultimately, we quantified the relative distribution of Ca_V_3.1 in different subnuclei of the IO. We observed that approximately half (see Results) of the neurons in every analyzed subnuclei are immuno-positive, except for the dorsal cap of Kooij which shows a higher Ca_V_3.1 channels expression. Our results provide evidence that olivary neurons can be distinguished in two populations based on the presence or absence of Ca_V_3.1 channels in their membrane. Moreover, our data suggest that the oscillatory profile of olivary neurons depends on the density of Ca_V_3.1 channels and this generates at least three different electrical behaviors, as predicted by Manor et al. (1997).

## Author Contributions

PB and MJ conceived and designed the study. PB conducted the experiments and analyzed all data. PB and MJ wrote, read and approved the manuscript.

## Conflict of Interest Statement

The authors declare that the research was conducted in the absence of any commercial or financial relationships that could be construed as a potential conflict of interest.

## References

[B1] Bazzigaluppi P. De Gruijl J. R. van der Giessen R. S. Khosrovani S. De Zeeuw C. I. de Jeu M. T. (2012a). Olivary subthreshold oscillations and burst activity revisited. *Front. Neural Circuits* 6:91 10.3389/fncir.2012.00091 PMC3504313 23189043

[B2] Bazzigaluppi P. Ruigrok T. Saisan P. De Zeeuw C. I. de Jeu M. (2012b). Properties of the nucleo-olivary pathway: an in vivo whole-cell patch clamp study. *PLoS ONE* 7:e46360 10.1371/journal.pone.0046360 PMC3459892 23029495

[B3] Choe W. Messinger R. B. Leach E. Eckle V. S. Obradovic A. Salajegheh R. (2011). TTA-P2 is a potent and selective blocker of T-type calcium channels in rat sensory neurons and a novel antinociceptive agent. *Mol. Pharmacol.* 80 900–910. 10.1124/mol.111.073205 21821734 PMC3198916

[B4] Choi S. Yu E. Kim D. Urbano F. J. Makarenko V. Shin H. S. (2010). Subthreshold membrane potential oscillations in inferior olive neurons are dynamically regulated by P/Q- and T-type calcium channels: a study in mutant mice. *J. Physiol.* 588 3031–3043. 10.1113/jphysiol.2009.184705 20547676 PMC2956943

[B5] Chorev E. Manor Y. Yarom Y. (2006). Density is destiny–on [corrected] the relation between quantity of T-type Ca2+ channels and neuronal electrical behavior. *CNS Neurol. Disord. Drug Targets* 5 655–662. 10.2174/187152706779025517 17168749

[B6] Chorev E. Yarom Y. Lampl I. (2007). Rhythmic episodes of subthreshold membrane potential oscillations in the rat inferior olive nuclei in vivo. *J. Neurosci.* 27 5043–5052. 10.1523/JNEUROSCI.5187-06.2007 17494690 PMC6672369

[B7] Courville J. Faraco-Cantin F. (1978). On the origin of the climbing fibers of the cerebellum. An experimental study in the cat with an autoradiographic tracing method. *Neuroscience* 3 797–809. 10.1016/0306-4522(78)90032-5 714252

[B8] Crunelli V. Lightowler S. Pollard C. E. (1989). A T-type Ca2+ current underlies low-threshold Ca2+ potentials in cells of the cat and rat lateral geniculate nucleus. *J. Physiol.* 413 543–561. 10.1113/jphysiol.1989.sp017668 2557441 PMC1189115

[B9] De Gruijl J. R. Bazzigaluppi P. de Jeu M. T. De Zeeuw C. I. (2012). Climbing fiber burst size and olivary sub-threshold oscillations in a network setting. *PLoS Comput. Biol.* 8:e1002814 10.1371/journal.pcbi.1002814 PMC3521668 23271962

[B10] De Zeeuw C. I. Chorev E. Devor A. Manor Y. Van Der Giessen R. S. De Jeu M. T. (2003). Deformation of network connectivity in the inferior olive of connexin 36-deficient mice is compensated by morphological and electrophysiological changes at the single neuron level. *J. Neurosci.* 23 4700–4711. 12805309 10.1523/JNEUROSCI.23-11-04700.2003 PMC6740782

[B11] Desclin J. C. (1974). Histological evidence supporting the inferior olive as the major source of cerebellar climbing fibers in the rat. *Brain Res.* 77 365–384. 10.1016/0006-8993(74)90628-3 4136782

[B12] Devor A. Yarom Y. (2002a). Coherence of subthreshold activity in coupled inferior olivary neurons. *Ann. N. Y. Acad. Sci.* 978:508 10.1111/j.1749-6632.2002.tb07594.x 12582080

[B13] Devor A. Yarom Y. (2002b). Generation and propagation of subthreshold waves in a network of inferior olivary neurons. *J. Neurophysiol.* 87 3059–3069. 12037208 10.1152/jn.2002.87.6.3059

[B14] Dreyfus F. M. Tscherter A. Errington A. C. Renger J. J. Shin H. S. Uebele V. N. (2010). Selective T-type calcium channel block in thalamic neurons reveals channel redundancy and physiological impact of I(T)window. *J. Neurosci.* 30 99–109. 10.1523/JNEUROSCI.4305-09.2010 20053892 PMC2880440

[B15] Horn K. M. Deep A. Gibson A. R. (2013). Progressive limb ataxia following inferior olive lesions. *J. Physiol.* 591 5475–5489. 10.1113/jphysiol.2012.234898 23027819 PMC3853490

[B16] Huguenard J. R. (1996). Low-threshold calcium currents in central nervous system neurons. *Annu. Rev. Physiol.* 58 329–348. 10.1146/annurev.ph.58.030196.001553 8815798

[B17] Khosrovani S. Van Der Giessen R.S. De Zeeuw C.I. De Jeu M.T. (2007). In vivo mouse inferior olive neurons exhibit heterogeneous subthreshold oscillations and spiking patterns. *Proc. Natl. Acad. Sci. U.S.A.* 104 15911–15916. 10.1073/pnas.0702727104 17895389 PMC2000380

[B18] Kim D. Song I. Keum S. Lee T. Jeong M. J. Kim S. S. (2001). Lack of the burst firing of thalamocortical relay neurons and resistance to absence seizures in mice lacking alpha(1G) T-type Ca(2+) channels. *Neuron* 31 35–45. 10.1016/S0896-6273(01)00343-9 11498049

[B19] Lampl I. Yarom Y. (1997). Subthreshold oscillations and resonant behavior: two manifestations of the same mechanism. *Neuroscience* 78 325–341. 10.1016/S0306-4522(96)00588-X 9145790

[B20] Lee S. Han T. H. Sonner P. M. Stern J. E. Ryu P. D. Lee S. Y. (2008). Molecular characterization of T-type Ca(2+) channels responsible for low threshold spikes in hypothalamic paraventricular nucleus neurons. *Neuroscience* 155 1195–1203. 10.1016/j.neuroscience.2008.06.055 18657597

[B21] Lefler Y. Yarom Y. Uusisaari M. Y. (2014). Cerebellar inhibitory input to the inferior olive decreases electrical coupling and blocks subthreshold oscillations. *Neuron* 81 1389–1400. 10.1016/j.neuron.2014.02.032 24656256

[B22] Leznik E. Llinas R. (2005). Role of gap junctions in synchronized neuronal oscillations in the inferior olive. *J. Neurophysiol.* 94 2447–2456. 10.1152/jn.00353.2005 15928056

[B23] Llinas R. Walton K. Hillman D. E. Sotelo C. (1975). Inferior olive: its role in motor learning. *Science* 190 1230–1231. 10.1126/science.128123 128123

[B24] Llinas R. Yarom Y. (1981). Electrophysiology of mammalian inferior olivary neurones in vitro. Different types of voltage-dependent ionic conductances. *J. Physiol.* 315 549–567. 10.1113/jphysiol.1981.sp013763 6273544 PMC1249398

[B25] Llinas R. Yarom Y. (1986). Oscillatory properties of guinea-pig inferior olivary neurones and their pharmacological modulation: an in vitro study. *J. Physiol.* 376 163–182. 10.1113/jphysiol.1986.sp016147 3795074 PMC1182792

[B26] Long M. A. Deans M. R. Paul D. L. Connors B. W. (2002). Rhythmicity without synchrony in the electrically uncoupled inferior olive. *J. Neurosci.* 22 10898–10905. 12486184 10.1523/JNEUROSCI.22-24-10898.2002 PMC2834587

[B27] Manor Y. Rinzel J. Segev I. Yarom Y. (1997). Low-amplitude oscillations in the inferior olive: a model based on electrical coupling of neurons with heterogeneous channel densities. *J. Neurophysiol.* 77 2736–2752. 9163389 10.1152/jn.1997.77.5.2736

[B28] Mathy A. Ho S. S. Davie J. T. Duguid I. C. Clark B. A. Hausser M. (2009). Encoding of oscillations by axonal bursts in inferior olive neurons. *Neuron* 62 388–399. 10.1016/j.neuron.2009.03.023 19447094 PMC2777250

[B29] McCormick D. A. Bal T. (1997). Sleep and arousal: thalamocortical mechanisms. *Annu. Rev. Neurosci.* 20 185–215. 10.1146/annurev.neuro.20.1.185 9056712

[B30] McKay B. E. McRory J. E. Molineux M. L. Hamid J. Snutch T. P. Zamponi G. W. (2006). Ca(V)3 T-type calcium channel isoforms differentially distribute to somatic and dendritic compartments in rat central neurons. *Eur. J. Neurosci.* 24 2581–2594. 10.1111/j.1460-9568.2006.05136.x 17100846

[B31] Mullen R. J. Buck C. R. Smith A. M. (1992). NeuN, a neuronal specific nuclear protein in vertebrates. *Development* 116 201–211. 1483388 10.1242/dev.116.1.201

[B32] Park Y. G. Park H. Y. Lee C. J. Choi S. Jo S. Choi H. (2010). Ca(V)3.1 is a tremor rhythm pacemaker in the inferior olive. *Proc. Natl. Acad. Sci. U.S.A.* 107 10731–10736. 10.1073/pnas.1002995107 20498062 PMC2890811

[B33] Perez-Reyes E. (2003). Molecular physiology of low-voltage-activated t-type calcium channels. *Physiol. Rev.* 83 117–161. 10.1152/physrev.00018.2002 12506128

[B34] Placantonakis D. G. Bukovsky A. A. Aicher S. A. Kiem H. P. Welsh J. P. (2006). Continuous electrical oscillations emerge from a coupled network: a study of the inferior olive using lentiviral knockdown of connexin36. *J. Neurosci.* 26 5008–5016. 10.1523/JNEUROSCI.0146-06.2006 16687492 PMC6674237

[B35] Shipe W. D. Barrow J. C. Yang Z. Q. Lindsley C. W. Yang F. V. Schlegel K. A. (2008). Design, synthesis, and evaluation of a novel 4-aminomethyl-4-fluoropiperidine as a T-type Ca2+ channel antagonist. *J. Med. Chem.* 51 3692–3695. 10.1021/jm800419w 18540666

[B36] Srinivas M. Calderon D. P. Kronengold J. Verselis V. K. (2006). Regulation of connexin hemichannels by monovalent cations. *J. Gen. Physiol.* 127 67–75. 10.1085/jgp.200509397 16380444 PMC2151478

[B37] Talley E. M. Cribbs L. L. Lee J. H. Daud A. Perez-Reyes E. Bayliss D. A. (1999). Differential distribution of three members of a gene family encoding low voltage-activated (T-type) calcium channels. *J. Neurosci.* 19 1895–1911. 10066243 10.1523/JNEUROSCI.19-06-01895.1999 PMC6782581

[B38] Urbano F. J. Simpson J. I. Llinas R. R. (2006). Somatomotor and oculomotor inferior olivary neurons have distinct electrophysiological phenotypes. *Proc. Natl. Acad. Sci. U.S.A.* 103 16550–16555. 10.1073/pnas.0607888103 17050678 PMC1616941

[B39] Van Der Giessen R. S. Koekkoek S. K. van Dorp S. De Gruijl J. R. Cupido A. Khosrovani S. (2008). Role of olivary electrical coupling in cerebellar motor learning. *Neuron* 58 599–612. 10.1016/j.neuron.2008.03.016 18498740

[B40] Van Der Giessen R. S. Maxeiner S. French P. J. Willecke K. De Zeeuw C. I. (2006). Spatiotemporal distribution of Connexin45 in the olivocerebellar system. *J. Comp. Neurol.* 495 173–184. 10.1002/cne.20873 16435305

[B41] Welsh J. P. Lang E. J. Sugihara I. Llinas R. (1995). Dynamic organization of motor control within the olivocerebellar system. *Nature* 374 453–457. 10.1038/374453a0 7700354

[B42] Wolpert D. M. Miall R. C. Kawato M. (1998). Internal models in the cerebellum. *Trends Cogn. Sci.* 2 338–347. 10.1016/S1364-6613(98)01221-2 21227230

[B43] Zhan X. Graf W. M. (2012). Harmaline attenuates voltage–sensitive Ca(2+) currents in neurons of the inferior olive. *J. Pharm. Pharm. Sci.* 15 657–668. 10.18433/J3W595 23331904

