# Supplementary figures and images for: Heterogeneous Expression of T-type Ca2+ Channels Defines Different Neuronal Populations in the Inferior Olive of the Mouse
Source: Front Cell Neurosci. 2016 Aug 4;10:192. doi: 10.3389/fncel.2016.00192 (PMC4972830; doi:10.3389/fncel.2016.00192)

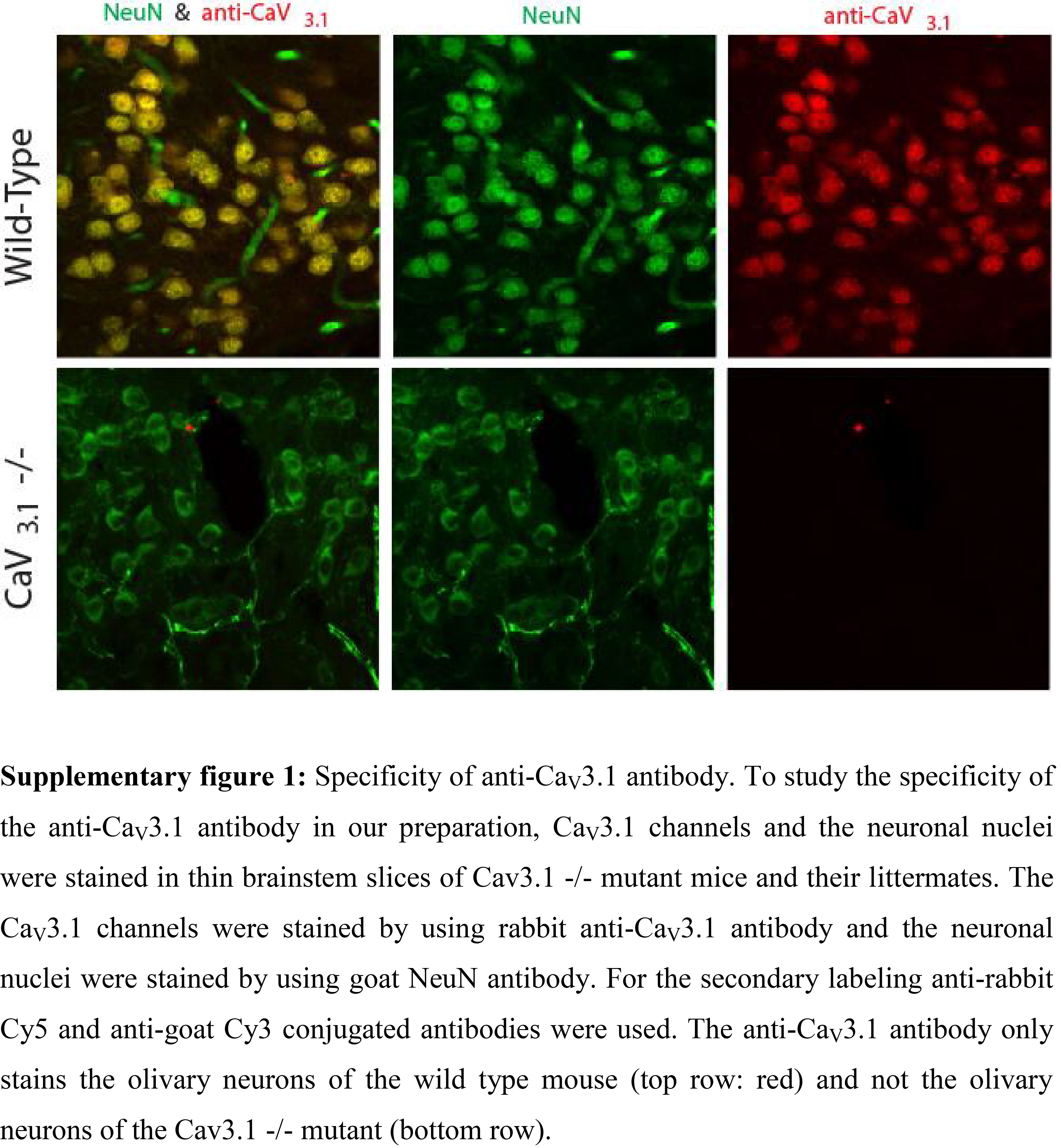

Supplement: Supplementary file 1 [file Image_1.JPEG]
